# Advances in CXCR7 Modulators

**DOI:** 10.3390/ph13020033

**Published:** 2020-02-21

**Authors:** Nicole Lounsbury

**Affiliations:** Department of Pharmaceutical Sciences, Larkin University College of Pharmacy, Miami, FL 33169, USA; nlounsbury@ularkin.org; Tel.: +1-305-760-74

**Keywords:** CXCR7, CXCL12, CXCR4

## Abstract

CXC chemokine receptor 7 (CXCR7) is a G-protein-coupled receptor that signals through the β-arrestin pathway. Its ligands include interferon-inducible T cell α chemoattractant (CXCL11) and stromal cell-derived factor-1 (CXCL12). It interacts with CXCR4, and the two are associated with various cancers, as well as other disease states such as coronary artery disease, stroke, inflammation and human immunodeficiency virus (HIV). Antibodies and small interfering RNA (siRNA) have shown the utility of antagonists of CXCR7 in these disease states. Although some small molecules were initially reported as antagonists due to their displayed activity, many function as agonists while still producing the desired pharmacologic effects. A potential reason for this contradiction is that effects may be due to elevated extracellular CXCL12 levels.

## 1. Introduction

CXC chemokine receptor 7 (CXCR7) is a G protein-coupled receptor (GPCR), also known as atypical chemokine receptor 3 (ACKR3). This receptor was an orphan receptor (RDC1) until 2007 when its two natural ligands, interferon-inducible T cell α chemoattractant (CXCL11) and stromal cell-derived factor-1 (SDF-1 or CXCL12), were identified [1,2]. CXCL11 is induced by interferon-γ in human microvascular endothelial cells (HMEC-1), hepatocytes and hepatic stellate cells in liver inflammation [3]. CXCL12 is involved in stem cell survival, proliferation and homing [4,5]. Other identified ligands include Dickkopf-3 (Dkk3) [6], viral CC motif chemokine 2/viral macrophage inflammatory protein II (vCCL2/vMIP-II) [7], adrenomedullin [8], BAM22 [9], and macrophage migration inhibitory factor (MIF) [10]. Dkk3 is a cytokine which leads to smooth muscle cell differentiation and endothelial repair [6]. vCCL2/vMIP-II is a chemokine from human herpesvirus-8 [7], while BAM22 is a peptide that is involved in regulating circadian glucocorticoid oscillation [9]. The interaction of MIF with CXCR7 is involved in platelet survival [10]. 

**Figure 1:**
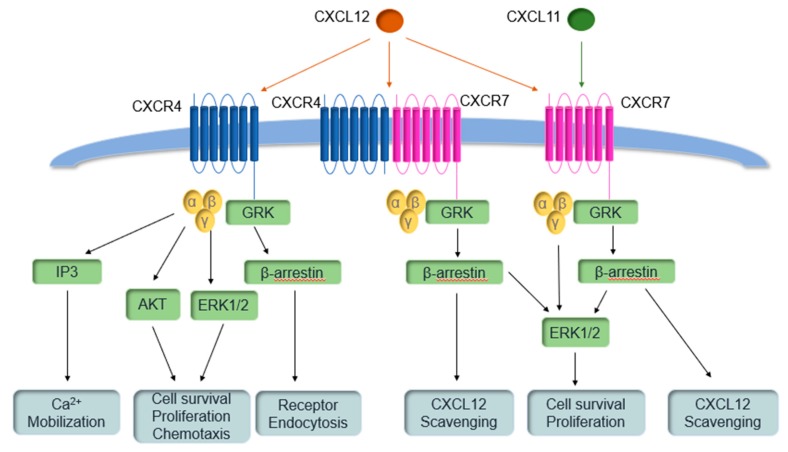
CXC chemokine receptor 4 (CXCR4) and CXCR7 pathways.

One of the unique aspects of CXCR7 is that it does not signal through a G protein-mediated pathway, but instead through the β-arrestin pathway [11]. CXCR7 does not cause the typical GPCR mobilization of calcium but binding of CXCL12 to CXCR7 leads to phosphorylation of Erk 1/2 [12,13]. CXCL12 is additionally a ligand for the CXCR4 receptor, although CXCL12 binds at a lower affinity to CXCR4 than CXCR7 [14]. The two receptors interact, as CXCR7 can dimerize with CXCR4 in order to decrease CXCR4 calcium signaling through rearrangement of the CXCR4/G protein complex [15]. In addition, inhibition of CXCR4 leads to an increase in CXCR7 levels [14,16]. Inhibition of CXCR7 does not affect CXCR4 levels, but CXCR7 can still modulate CXCR4 signaling through dimerization even when CXCR7 is inhibited [15]. Overall, the two proteins affect cell survival and proliferation as well as chemotaxis [13]. The pathways for CXCR7 and CXCR4 are illustrated in Figure 1. As well as forming heterodimers with CXCR4, CXCR7 forms homodimers [17]. The function of CXCR7 differs by cell type; it has been proposed that CXCR7 is a scavenger for CXCL12, thus affecting the CXCL12 gradient and modulating CXCR4 signaling [14]. High expression of CXCR7 is observed in monocytes and mature B cells [18], and there is a correlation between the levels of the protein at the plasma membrane and the survival and differentiation of B cells [19]. CXCR7 is also expressed in the mesenchyme and microvasculature of the heart valve and ventricular septum, and the absence of CXCR7 is lethal in C57BL/6 mice, who lack CXCL11, due to defects in these areas [20]. CXCR7 is furthermore expressed in neuronal tissue [18] and is involved in embryonic development [20], directional cell migration [21,22] and immune functions [23]. Cooperatively, CXCR4 and CXCR7 regulate progenitor cell homing [24] and tissue and interneuron migration [25,26,27,28]. 

## 2. The Physiological Roles of CXCR7

CXCR4 and/or CXCR7, with their ligand CXCL12, are associated with many neurological and inflammatory conditions, as well as many cancers. CXCR7 is up-regulated in disease states including post-ischemic stroke [29,30], multiple sclerosis [31], Alzheimer’s disease [32], epilepsy [33], rheumatoid arthritis [34], autism [35], and coronary artery disease [36]. Increased expression is also observed in many cancers, including prostate [16], pancreatic [37], ovarian [38], colon [39], kidney [40], liver [40], lung and breast [41] and CXCR7 is involved in the growth, metastasis and survival of these tumor cell lines. The receptor additionally functions as a coreceptor for various human immunodeficiency virus (HIV) strains [42]. As CXCR7 is up-regulated in a majority of these disease states, antibodies and/or small interfering RNA (siRNA) have been used as methods to inhibit CXCR7. The successful disease amelioration by these antibodies and siRNA implicates antagonists of CXCR7 as a potential pharmacological treatment option. 

### 2.1. The Role of CXCR7 in Neurological Conditions

The expression of CXCR7 and its ligand CXCL12 are increased post-ischemic stroke [29,30], as CXCL12 and CXCR7 have neuroprotective effects [29]. CXCR4 is also up-regulated, but only CXCR7 was correlated to an increased survival in mouse neural progenitor cells (mNPCs). An anti-CXCR7 antibody was administered to post-ischemic rats, and enhanced neurogenesis and cognitive function [43]. In multiple sclerosis, the loss of CXCL12 occurs from abluminal surfaces in the CNS, while CXCR7 expression increases as a scavenger of CXCR12 [31]. In experimental autoimmune encephalomyelitis (EAE) models in rats, an animal model of MS, an increase in astrocytic CXCR7 expression in the spinal cord was observed [32]. This overexpression causes the chemotaxis of microglia and is correlated with disease severity [44]. An anti-CXCR7 antibody ameliorated the symptoms of EAE. Higher expression of CXCR7 is also detectable in the hippocampus of patients with Alzheimer’s disease as well as after spinal cord compression [32]. In a mouse model of epilepsy, as well as in tissues of temporal lobe epilepsy patients, CXCR7 is up-regulated in the hippocampal dentate gyrus region [33]. The receptor controls the expression of N-methyl-D-aspartate receptor subunit 2A (NR2A), consequently regulating seizures through N-methyl-D-aspartate receptor-(NMDAR)-mediated synaptic neurotransmission. Silencing of CXCR7 with short hairpin RNA (shRNA) lowered the susceptibility of epileptic mice to seizures.

### 2.2. The Role of CXCR7 in Inflammation

In rheumatoid arthritis, CXCR7 is expressed on endothelial cells in the synovium as well as on unstimulated human umbilical vein endothelial cells (HUVECs), and CXCR7 and CXCL12 are involved in angiogenesis [34]. CXCL12 is also up-regulated in the synovium and has pro-inflammatory effects in autoimmune arthritis through its activation of osteoclast differentation from splenocytes [45]. CXCR7 is up-regulated in the airway epithelium and involved in the regulation of allergic airway inflammation [46]. In acute pulmonary inflammation, CXCR7 expression is increased in the pulmonary epithelium and on polymorphonuclear neutrophils (PMNs) and is involved in transepithelial PMN migration [47]. The receptor is additionally up-regulated in obese adipose tissue, and adipocytes express CXCR7 in obesity-associated chronic inflammation [48]. An anti-CXCR7 antibody ameliorated insulin sensitivity and glucose tolerance in obese mice that were fed high-fat diets [49].

### 2.3. The Role of CXCR7 in Cancers

CXCR7 has been implicated in the growth, metastasis, and survival of tumor cell lines such as prostate [16], glioma [12], bladder [50], pancreatic [37], Kaposi sarcoma [51], ovarian [38], cervical [52], colon [39], uterine [40], kidney [40], liver [40], stomach [40], lung and breast [41] as well as hepatocellular carcinoma [3]. CXCR7 is present on tumor endothelial, glioma and microglial cells, and is often colocalized with CXCL12 [12]. In addition, CXCR7 is expressed on blood vessels that are associated with breast and lung cancer tumors but is not expressed on healthy blood vessels [41]. This specialized vascular localization may implicate CXCR7 in angiogenesis as well as tumor growth and metastasis. Expression of CXCR7 increases with increasing malignancy of tumors [12]. Its role in tumorigenesis in colorectal cancer is likely through histone demethylation via a CXCR4/CXCR7 heterodimer [53]. Overall, the overexpression of CXCR7 is significantly correlated to lowered overall survival, disease-free survival, recurrence-free survival and progression-free survival [54,55,56]. In solid tumors, high levels of CXCR7 predict a high risk of lymph node metastasis [55]. In gastric cancer, lipopolysaccharide induces the TLR4 (toll-like receptor 4)/MD-2 (myeloid differential protein-2) pathway, which leads to the upregulation of CXCR7, and ultimately tumor growth and metastasis [57]. CXCR7 regulates transforming growth factor-β1 (TGF-β1)/Smad2/3 signaling in head and neck squamous cell carcinoma [58]. Both the receptor and CXCL11 are present at higher levels in hepatocellular carcinoma (HCC) than in healthy livers in both mice and humans [3]. HCC tumors are under acidic and hypoxic conditions, and CXCR7 is up-regulated in these conditions in HMEC-1 cells. 

Antibodies or siRNA have been used as proof of concept for inhibition of CXCR7 in cancer cell lines. siRNA was used to inhibit CXCR7 in a prostate cancer cell line and tumor growth in vitro was suppressed [16]. The same effect was observed in oral squamous cell carcinoma treated in vitro with micro RNA (miRNA) [59], and in an in vitro renal cell carcinoma cells under hypoxia model treatment with miRNA reduced viability, invasion ability and migration ability [60]. Suppression of growth was also seen in vitro and in vivo using miRNA in an esophageal cancer cell line [61]. Similar delaying of growth of tumors was observed in a model of endometrial carcinoma [62] and esophageal cancer [63] in mice with siRNA, as well as inhibition of invasion and proliferation of glioma cells [64]. Mice with glioblastoma which were treated with an antibody against CXCR7, in addition to temozolomide, displayed increased overall survival and reduced tumor size [65]. CXCR7-targeting nanobodies also inhibited tumor growth in a mouse model of head and neck cancer [66]. Andrographolide decreases CXCR7 expression, which may be part of its mechanism of action of inhibition of prostate cancer cell viability [67]. 

### 2.4. The Role of CXCR7 in Other Disease States

Immune alterations occur in autism spectrum disorders, and CXCR7 is up-regulated in both peripheral blood mononuclear cells (PBMCs) and CD4^+^ T cells in children with autism [35]. CXCR7 is also up-regulated in endometriosis and expressed throughout vascular smooth muscle cells and endothelial cells [68]. Overexpression of CCXR7 is observed in animal models of pulmonary fibrosis in endothelial cells [69]. CXCR7 attenuates the endothelial-to-mesenchymal transition (EndMT) and may regulate TGF-β-induced EndMT. CXCR7 is a coreceptor for numerous human immunodeficiency virus type 1 (HIV-1), HIV-2 and simian immunodeficiency virus (SIV) strains [42]. 

In cardiac disorders, CXCR7, CXCR4 and CXCL12 expression in platelets affects the disease severity of coronary artery disease [36]. Specifically, CXCR7 and CXCL12 function in lipid uptake, thus causing a thrombogenic effect on platelets. CXCR7 is also induced, and mediates CXCL11 and CXCL12, in monocyte-to-macrophage differentiation, and causes increased macrophage phagocytosis, which can lead to atherogenesis [70]. Two agents which lower the expression of CXCR7 have implications in the treatment of atherosclerosis, which are atorvastatin [71] and pioglitazone [72].

In the previously mentioned disease states, as CXCR7 is overexpressed and as antibodies and siRNA/miRNA showed disease ameliorating results, CXCR7 antagonists are indicated as a novel therapeutic option. There are a relatively small number of CXCR7 modulators disclosed to date, but most are peptide-like, possess only moderate potency for CXCR7 and none have advanced to clinical trials. 

## 3. CXCR7 Modulators

### 3.1. Small Molecule Modulators

**Figure 2:**
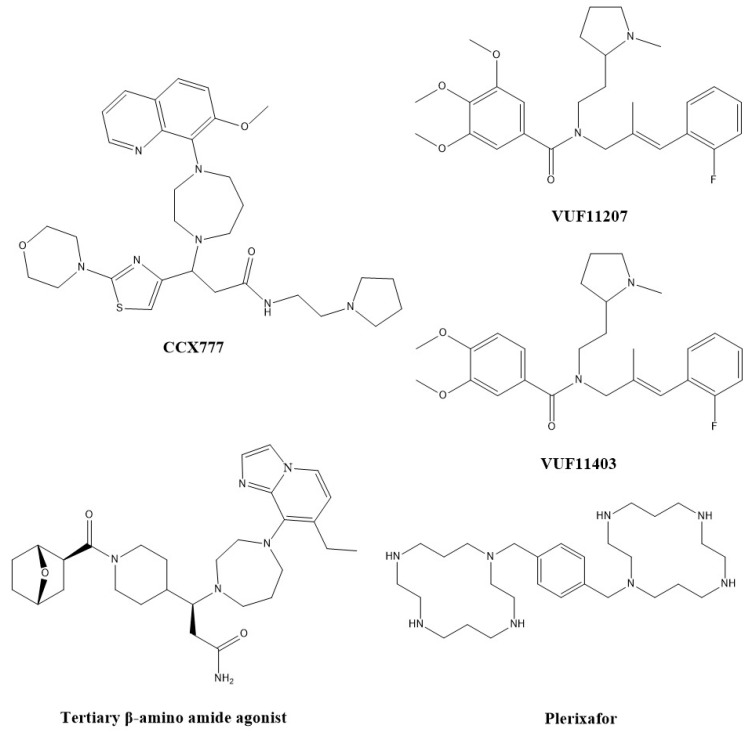
Small molecule modulators of CXCR7.

Based on the hypothesis of the utility of CXCR7 antagonists in various disease states, several antagonists were developed. ChemoCentryx reported inhibitors, including CCX754, CCX733, CCX771 (structures not disclosed) and CCX777 (Figure 2), which were identified in manuscripts as ligands which did not lead to the phosphorylation of ERK or Akt [73]. CCX771, identified as an antagonist, was given to mice with experimental autoimmune encephalomyelitis (EAE), a model of MS, and reduced the disease severity while ameliorating symptoms [31]. CCX771 further halted the proliferation and invasion of glioma cells, similar to results that were seen with siRNA, and was again reported as an antagonist [64]. CCX771 also prolonged survival and led to tumor regression and inhibition of tumor recurrence in rodent models of glioblastoma multiforme after irradiation [74]. In a mouse model of hyperlipidemia, CCX771 lowered circulating very-low density lipoprotein levels, thus protecting against atherosclerosis [75]. The compound also demonstrated utility in a mouse model of endometriosis by reducing the size of lesions through its effects on bone marrow cell engraftment [76]. However, it was additionally reported as an agonist that recruited β-arrestin-2 to CXCR7 and was shown to block the transendothelial migration of CXCR4^+^/CXCR7^+^ human cancer cells, a similar result seen with CXCL11 [77]. 

Similarly, CCX733 was first reported as an antagonist, and inhibited CXCL12-induced angiogenesis in HUVECs [34]. Similar results were seen with a CXCR4 antagonist (AMD3100) and an anti-CXCR4 antibody. In addition, in a mouse model of rheumatoid arthritis, CCX733 lowered the clinical arthritis scores and prevented joint destruction. CCX733 also decreased the antiapoptotic effects of CXCL12 in glioma cells [12]. Similar to CCX771, CCX733 was later reported as an agonist in that it leads to CXCR7 homodimerization, similar to CXCL11 and CXCL12 [17]. CCX733 reduced the expression level of CXCR4 and thus the sensitivity of cells to CXCL12, and ultimately inhibited CXCL12-induced HUVEC tube formation [78]. However, the inhibition of HUVEC tube formation was not displayed with an antagonistic antibody, thus demonstrating its likely agonistic effects. 

Other analogs include CCX754. CCX754 was evaluated as an antagonist in immunodeficient or syngeneic mice that were engrafted with human lung carcinoma A549 or mouse lung carcinoma LL/2, both of which express CXCR7 [2]. In both models, tumor size was reduced upon treatment with a CXCR7 inhibitor. As in the previous two cases, CCX754 has also been defined as an agonist that causes CXCR7 homodimerization [17]. A final analog, CCX777 (Figure 2), acts as a partial agonist according to dose-response curves [79]. 

Witjmans et al. [80] performed structure-activity relationships on the styrene-amide scaffold from the original Chemocentryx compounds, and discovered 2 CXCR7 agonists (VUF11207 and VUF11403, Figure 2) with low nM EC_50_ values. They induce the recruitment of β-arrestin-2 and internalization of CXCR7. 

A 1,4-diazepene agonist was discovered with low nM potency, but it is lipophilic and inhibits hERG [81]. After performing SAR, a tertiary β-amino amide (Figure 2) was developed [82]. This compound has similar affinity for CXCR7 (a K_i_ of 13 nM and an EC_50_ of 11 nM for β-arrestin recruitment), and a larger therapeutic window for hERG. It demonstrated a reduction of cardiac fibrosis in a model of isoproterenol-induced cardiac injury in mice.

Plerixafor (Figure 2), a bicyclam, is an antagonist for CXCR4 that is used clinically to mobilize hematopoietic stem cells in multiple myeloma and non-Hodgkin’s lymphoma in combination with granulocyte-colony stimulating factor [83]. Plerixafor also inhibits the proliferation and invasiveness of tumor cell models such as glioblastoma multiforme cells. In contrast to its role with CXCR4, plerixafor is an allosteric agonist of CXCR7, as it recruits β-arrestin with an EC_50_ of 140 µM [84].

Yoshikawa et al. [85] discovered a series of 21 CXCR7 antagonists that block CXCL12 binding with μM IC_50_ values using homology modeling built on the few known CXCR7 and CXCR4 inhibitor scaffolds. Two of their reference compounds were micromolar ligands from the ChemDiv libraries, while another two were nanomolar ligands from patents. These compounds did not show significant inhibition of CXCR4 at 10 μM. Functional activity assays were not reported, and it is possible that they are agonists as all other reported compounds are.

### 3.2. Peptide-Based Modulators

**Figure 3:**
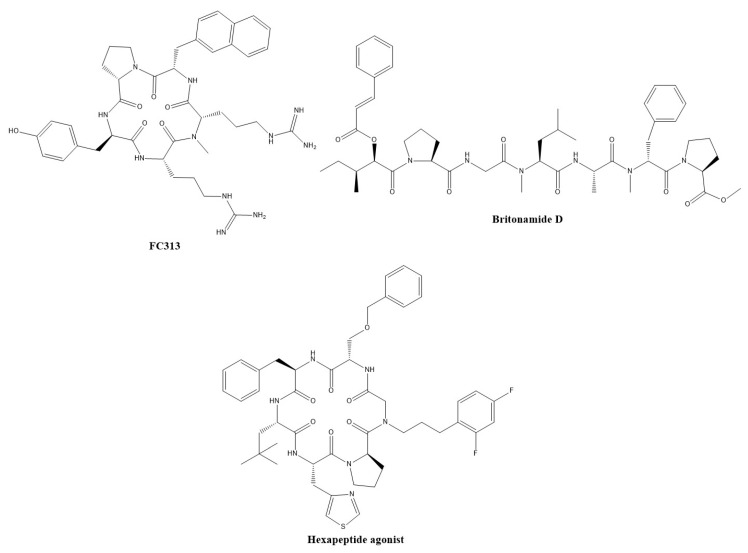
Peptide-based modulators of CXCR7.

Peptide-based agonists at CXCR7 have also been developed. FC313 is a cyclic pentapeptide (Figure 3) that is a selective agonist for CXCR7 [86], which was developed from FC131, a cyclic pentapeptide CXCR4 antagonist [87]. The pentapeptides contain a phenol, two guanidines and a naphthalene. FC313 had an IC_50_ value of 0.80 µM for CXCL12 binding, with no significant binding to CXCR4, while the EC_50_ for recruitment of β-arrestin was 0.095 µM. Meanwhile, the EC_50_ for CXCL12 is 0.014 µM. Further SAR studies of FC313 led to the development of a more potent pentapeptide ligand, which has an IC_50_ value of 0.17 µM for CXCL12 binding (while that for CXCR4 was 17 µM) and an EC_50_ for recruitment of β-arrestin of 0.49 µM [88]. 

Five britonamides, which are linear peptides, were isolated from a marine cyanobacterial sample, and two other synthetic analogs were created [89]. While screening one of the brintonamides in a GPCR assay (agonist and antagonist), it was found that while it displayed antagonistic activity at four other GPCRs (CCR10, TACR2, SSTR3, OXTR), it only displayed agonistic activity at CXCR7. After screening all seven of the britonamides at this target, four of them had µM EC_50_ values as agonists of CXCR7. The most potent ligand of CXCR7, britonamide D (Figure 3), halted the proliferation and migration of a cellular model of breast cancer, but the authors hypothesized that this was due to inhibition of CCR10, another chemokine receptor. 

A screen of 17 mer peptides, which contained the amino acids of wild-type CXCL12 at positions 5–17 and randomized amino acids at 1–4, identified two peptides as allosteric CXCR4 agonists [90]. Further studies of related 17 mer peptides led to a peptide with GSLW at amino acid positions 1–4 which functioned as an allosteric modulator of CXCR7 that activated signaling to β-arrestin-2. 

Boehm et al. [91] performed SAR on a macrocyclic hexapeptide modulator from the patent literature [92], with low nM functional activity, although it possessed a µM binding affinity and poor permeability. Modifications led to the development of a peptide agonist (Figure 3) with improved potency (EC_50_ = 15 nM) and permeability.

TC14012, a cyclic peptide, is an antagonist for CXCR4 but an agonist for CXCR7, with an EC_50_ for recruitment of β-arrestin of 350 nM [93]. Resembling plerixafor, TC14012 inhibits the migration and apoptosis of tumor cell lines such as chronic lymphocytic leukemia B cells [94]. The mode of binding for both to CXCR7 is similar to the binding of CXCR4 antagonists [95]. TC14012 reduced fibrosis and lead to alveolar repair in mice with repeated lung injuries [96]. TC14012 has further shown activity as a promoter of angiogenesis through endothelial progenitor cells (EPCs) in diabetic limb ischemia, likely through its agonistic effects at CXCR7 [97]. 

CXCR7 modulators and inhibitors were also identified in a number of patents [98,99,100,101,102,103,104,105,106,107,108,109,110,111]. 

## 4. Why Agonists are Producing Hypothesized Antagonist Results 

Although several modulators of CXCR7 have been discovered, many do not have reported pharmacological effects. The Chemocentryx compounds, however, were used in various disease state models, and were likely first reported as antagonists due to the physiological results that they produced. There may be several reasons why agonists display the physiological activity that was hypothesized to be exhibited by antagonists. The mechanism of action for agonists or antagonists may be more related to CXCL12-mediated effects than CXCR7. CXCL12 levels are increased in most pathological conditions that CXCR7 is overexpressed in, including tumors [112,113], rheumatoid arthritis [34,114,115], stroke [116,117], multiple sclerosis [118], traumatic brain injury [119,120,121] and obesity [48]. An exception is that decreased blood levels of CXCL12 are observed in Alzheimer’s disease [122]. In tumor cells, CXCL12 induces transendothelial migration, and thus the metastatic spread of CXCR4^+^CXCR7^+^ cells [123]. CXCR7 agonists lead to internalization of the receptor, thus blocking it from binding to CXCL12 [17]. This leads to increased extracellular levels of CXCL12, which may be responsible for the pathophysiological effects observed with CXCR7 agonists [31]. Further highlighting this theory is results that an anti-CXCL12 antibody suppressed tumor growth and/or metastasis in various tumor models [124]. In addition, a chalcone CXCL12 neutraligand displayed anti-inflammatory properties in an allergic airway hypereosinophilia murine model [125]. Antibodies against CXCR7 display similar results, hence accounting for the similarity between treatment with CXCR7 agonists and antibodies [75]. However, in one case although CXCR7 agonists inhibited CXCL12-induced angiogenesis, this result was not seen with an anti-CXCR7 antibody [78]. Initial studies of CXCR7 used antibodies that were later revealed to be nonspecific for CXCR7 [16,126,127,128,129], which may be responsible for some of the contradicting results in the foundational work, although recent antibodies are demonstrated to be specific for the target. Although nanobodies were only used in one study of tumor vascularization in head and neck cancer, the anti-CXCR7 nanobodies are inverse agonists [66]. Other modulators may be inverse agonists, but no other data has supported this thus far.

Another possible mechanism of action for the agonists is the downregulation of CXCR4, as CXCR7 signaling accomplishes this result [78]. It is also possible that prolonged exposure to an agonist causes desensitization, and thus loss of function of CXCR7 [62].

## 5. Conclusions

CXCR7 is associated with many disease states such as cancer, coronary artery disease, stroke, inflammatory conditions and HIV. Antibodies and siRNA targeted to CXCR7 have demonstrated the utility of antagonists to treat these diseases and highlight its value as a novel target. The first small molecule modulators discovered were identified as antagonists but have since been revealed to be agonists. Following this small molecule agonists were discovered, as well as peptide-based agonists. Therefore, most of the modulators discovered are agonists, although they display the physiological activity seen with antibodies and siRNA and expected out of antagonists. The physiological effects may be through increasing CXCL12 levels, or desensitization of the receptor or the downregulation of CXCR4 could also cause the same effects. As this GPCR is a novel target for many disease states, future research is needed as to whether agonism is required for disease-ameliorating effects, as well as whether the effects are truly through CXCR7 or instead possibly through CXCL12.
